# Metal Ion Activation of *Clostridium sordellii* Lethal Toxin and *Clostridium difficile* Toxin B

**DOI:** 10.3390/toxins8040109

**Published:** 2016-04-13

**Authors:** Harald Genth, Ilona Schelle, Ingo Just

**Affiliations:** Institute for Toxicology, Hannover Medical School, Carl-Neuberg-Str. 1, D-30625 Hannover, Germany; Ilona.Schelle@t-online.de (I.S.); just.ingo@mh-hannover.de (I.J.)

**Keywords:** glycosyltransferase, UDP-glucose hydrolysis, cytopathic effect, Rho, Ras, manganese, transepithelial resistance, small GTPases, Madin-Darby canine kidney (MDCK-C7) cells

## Abstract

Lethal Toxin from *Clostridium sordellii* (TcsL) and Toxin B from *Clostridium difficile* (TcdB) belong to the family of the “Large clostridial glycosylating toxins.” These toxins mono-O-glucosylate low molecular weight GTPases of the Rho and Ras families by exploiting UDP-glucose as a hexose donor. TcsL is casually involved in the toxic shock syndrome and the gas gangrene. TcdB—together with Toxin A (TcdA)—is causative for the pseudomembranous colitis (PMC). Here, we present evidence for the *in vitro* metal ion activation of the glucosyltransferase and the UDP-glucose hydrolysis activity of TcsL and TcdB. The following rating is found for activation by divalent metal ions: Mn^2+^ > Co^2+^ > Mg^2+^ >> Ca^2+^, Cu^2+^, Zn^2+^. TcsL and TcdB thus require divalent metal ions providing an octahedral coordination sphere. The EC_50_ values for TcsL were estimated at about 28 µM for Mn^2+^ and 180 µM for Mg^2+^. TcsL and TcdB further require co-stimulation by monovalent K^+^ (not by Na^+^). Finally, prebound divalent metal ions were dispensible for the cytopathic effects of TcsL and TcdB, leading to the conclusion that TcsL and TcdB recruit intracellular metal ions for activation of the glucosyltransferase activity. With regard to the intracellular metal ion concentrations, TcsL and TcdB are most likely activated by K^+^ and Mg^2+^ (rather than Mn^2+^) in mammalian target cells.

## 1. Introduction

Lethal Toxin (TcsL) from *Clostridium sordellii* and Toxin B from *Clostridium difficile* (TcdB) belong to the family of the “Large clostridial glycosylating toxins” (LCGTs). The LCGTs further encompass the hemorrhagic toxin (TcsH) from *C. sordellii*, toxin A (TcdA) from *C. difficile*, alpha-toxin (Tcnα) from *Clostridium novyi*, and the glycosylating toxin (TcpE) from *Clostridium perfringens* type E strains [1,2]. The LCGTs exhibit a multidomain structure harboring domains required for self-mediated cell entry. Thereby, the N-terminally located glycosyltransferase domain enters the target cell cytosol by receptor-mediated endocytosis (short trip uptake). The glycosyltransferase domain catalyzes the transfer of a glucose moiety or—in the cases of Tcnα and TcpE—of a *N*-acetylglucosamine moiety to small GTPases of the Rho and Ras subfamilies. In particular, TcsL glucosylates the Ras family GTPases (H/K/N/R) Ras, Rap1/2, and Ral, as well as the Rho family GTPases Rac and Cdc42, while TcdB specifically glucosylates Rho, Rac, and Cdc42 subfamily GTPases [3,4,5]. Mono-glucosylation at Thr-35/-37, a pivotal residue within the effector loop of Rho-/Ras-GTPases, prevents GTP-driven activation of Ras/Rho proteins, resulting in functional inactivation of the GTPases [6,7]. In cultured cells, substrate GTPase glucosylation by TcsL and TcdB results in a loss of cell-cell- and cell-matrix-interaction [8,9,10] and in cell death including upregulation and activation of the cell death-regulating GTPase RhoB [11,12,13,14]. TcsL-induced loss of cell-matrix adhesion, a hallmark of TcsL cytopathic activity, depends on disassembly of focal adhesion complexes rather than of actin depolymerization [9,15].

The 3D structures of TcdB and TcsL have led to their classification as type A glycosyltransferases [16,17]. The enzymatic activity of type A glycosyltransferases employing nucleotide hexoses depends on divalent metal ions. In the absence of their substrate GTPase, the LCGTs exhibit UDP-hexose hydrolysis activity. While the metal ion dependencies of UDP-glucose hydrolysis activity of TcdB and TcdA have been evaluated in some detail [18,19], data on the metal ion dependency of the glucosyltransferase activity of the LCGTs and of TcsL in particular are not available. We here show that the glucosyltransferase and the UDP-glucose hydrolysis activities of recombinant glucosyltransferase domains of TcsL (rN-TcsL) and of TcdB (rN-TcdB) are strongly stimulated by monovalent and divalent metal ions in cell-free systems.

## 2. Results and Discussion

### 2.1. Divalent Metal Ion Dependency of TcsL

**Figure 1:**
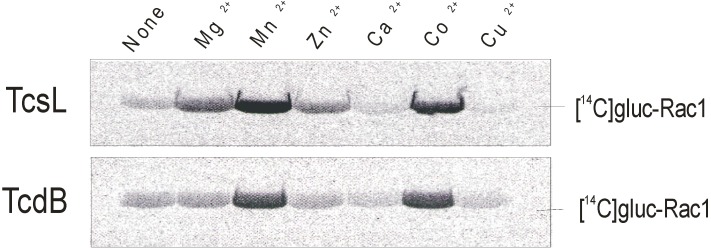
Divalent metal ion-activated glycosyltransferase activity of *C. sordellii* lethal toxin (TcsL). Rac1 (50 µg/mL) and rN-TcsL (0.3 nM) or rN-TcdB (0.3 nM) as indicated were incubated in the presence of the indicated divalent metal ions (1 mM) at 37 °C for 10 min. [^14^C]glucosylated Rac1 was separated by SDS-page and visualized by autoradiography.

The divalent metal ion requirement of the glucosyltransferase activity of TcsL was evaluated in a cell-free system using the recombinantly prepared the N-terminally located domain of TcsL (rN-TcsL) and Rac1 as substrate GTPase in the presence of UDP-[^14^C]glucose. Mn^2+^ turned out to be the most efficacious activator of rN-TcsL glucosyltransferase activity, whereas Co^2+^ and Mg^2+^ were partial activators (Figure 1). Zn^2+^ also stimulated rN-TcsL activity to some extent, whereas Ca^2+^ and Cu^2+^ did not. Remarkable transferase activity was observed in the absence of added divalent metal ions (none) due to the presence of Mg^2+^ required for the stability of the substrate GTPase. The analysis of divalent metal ion requirement of rN-TcsL was thus biased by the Mg^2+^ requirement of the substrate GTPase (Figure 1).

**Figure 2:**
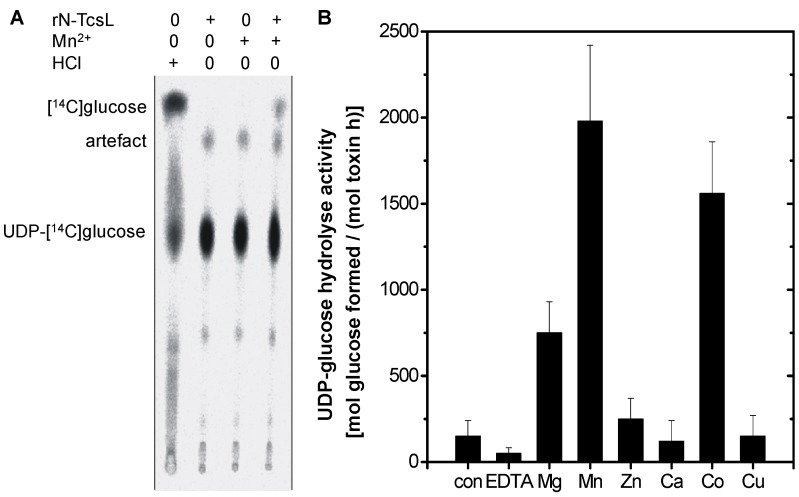
Divalent metal ion-activated UDP-glucose hydrolysis activity of *C. sordellii* lethal toxin (TcsL). (**A**) UDP-[^14^C]glucose (100 µM) was incubated in the presence of 0.1 M·HCl with Mn^2+^ (1 mM) and rN-TcsL (100 nM) as indicated at 37 °C for 30 min. UDP-[^14^C]glucose and [^14^C]glucose were separated by thin layer chromatography on PEI cellulose and visualized by autoradiography. (**B**) UDP-[^14^C]glucose (100 µM) was incubated in the presence of rN-TcsL (100 nM) with the indicated divalent metal ions (1 mM) at 37 °C for 30 min. Signal intensities of formed [^14^C]glucose were quantified and are given as means ± SD (*n* = 3).

In the absence of substrate GTPases, rN-TcsL catalyzes hydrolytic cleavage of UDP-glucose into UDP and glucose (UDP-glucose hydrolysis activity), which is exploited as a surrogate for the physiologically relevant glucosyltransferase activity [18,19,20]. In the absence of rN-TcsL, spontaneous hydrolysis in the presence of Mn^2+^ was barely detected at neutral pH, as analyzed upon thin layer chromatography (PEI-cellulose as matrix) for the separation of UDP-[^14^C]glucose (educt) and [^14^C]glucose (product) (Figure 2A). Proton-catalyzed hydrolysis of UDP-glucose into UDP and [^14^C]glucose was applied as control to prove that the spot assigned to glucose was in fact [^14^C]glucose (Figure 2A). Freshly prepared rN-TcsL exhibited only (if any) poor UDP-glucose hydrolysis activity in the presence of monovalent K^+^ (Figure 2A). This activity was strongly stimulated in the presence of several divalent metal ions (1 mM each), and the following rating was found (Figure 2B): Mn^2+^ > Co^2+^ > Mg^2+ ^> Zn^2+^, Ca^2+^, Cu^2+^. Thus, divalent metal ion requirement of glucosyltransferase (Figure 1) and UDP-glucose hydrolysis activity (Figure 2B) were almost comparable. In the presence of the chelator EDTA (1 mM), rN-TcsL-catalyzed UDP-glucose hydrolysis activity was almost completely suppressed, supporting the essential role of divalent metal ions in enzyme activity (Figure 2B). The finding that Ca^2+^ failed to activate both glucosyltransferase activity (Figure 1) and UDP-glucose hydrolysis activity (Figure 2B) is consistent with structural data showing that the active center of rN-TcsL is disassembled in the presence of Ca^2+^ (albeit Ca^2+^ being capable of mediating UDP-glucose binding to rN-TcsL) [16]. rN-TcsL glucosyltransferase activity and UDP-glucose hydrolysis activity thus seems to depend on divalent metal ions providing a strictly octahedral coordination sphere including Mn^2+^, Co^2+^, or Mg^2+^ (Figure 2B).

**Figure 3:**
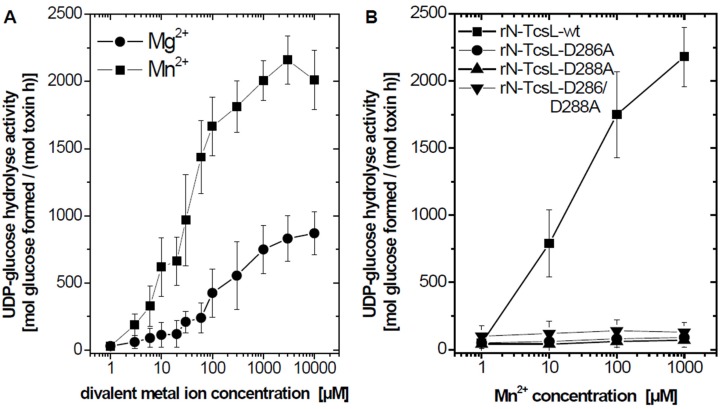
Divalent metal ion activation of rN-TcsL. A. UDP-[^14^C]glucose (100 **µ**M) was incubated in the presence of rN-TcsL (100 nM) with the indicated concentrations of Mn^2+^ or Mg^2+^ at 37 °C for 30 min. B. UDP-[^14^C]glucose (100 µM) was incubated in the presence of rN-TcsL (100 nM) and the indicated rN-TcsL mutants (1 µM) with the indicated concentrations of Mn^2+^ at 37 °C for 30 min. UDP-[^14^C]glucose and [^14^C]glucose were separated by thin layer chromatography on PEI cellulose and visualized by autoradiography. Signal intensities of formed [^14^C]glucose are quantified and given as mean ± SD (*n* = 3).

UDP-glucose hydrolysis activity of rN-TcsL was next analyzed in the presence of increasing concentrations of the most efficacious divalent metal activator Mn^2+^ and the partial divant metal activator Mg^2+^. The UDP-glucose hydrolysis activity *versus* divalent metal concentration curve exhibited a sigmoid shape (Figure 3A). The EC_50_ values were estimated to about 28 µM for Mn^2+^ and 180 µM for Mg^2+^ using the sigmoid E_max_ model. Mn^2+^ thus turned out to be the most efficacious divalent metal ion activator *in vitro*. Albeit being the most efficacious divalent metal ion activator *in vitro*, Mn^2+^ (as well as Co^2+^) may be excluded as intracellular activators of TcsL due to their low free intracellular concentrations of maximal 1 µM. rN-TcsL more likely meets activating levels of Mg^2+^, which free intracellular concentration is in the millimolar range [21].

### 2.2. Essential Role of the DxD Motif for Mn^2+^-Activated UDP-Glucose Hydrolysis Activity

The divalent metal-dependent coordination of UDP-hexoses within the catalytic cleft of all members of the LCGT family requires the Asp**-**any amino acid-Asp (DxD) motif [22]. From the 3D-structure of rN-TcsL, it can be deduced that Asp-288 directly binds to Mn^2+^, whereas Asp-286 binds the ribosyl and glucosyl moieties of UDP-glucose [16]. To further provide evidence on essential role of the DxD in Mn^2+^-activated UDP-glucose hydrolysis activity, the glycohydrolase activity was analyzed using several rN-TcsL versions with mutant DxD motif including rN-TcsL-D286A, rN-TcsL-D288A, as well as the double mutant rN-TcsL-D286A-D288A [20]. Exchange of either aspartate residue was sufficient to completely block Mn^2+^-activated UDP-glucose hydrolysis activity (Figure 3B). The DxD motif is thus essential for Mn^2+^-dependent UDP-glucose hydrolysis activity of TcsL. These observations are consistent with former data showing that either exchange of either Asp abolishes the rN-TcsL glucosyltransferase activity [20]. Divalent metal ion activation of rN-TcsL depends on the DxD motif.

### 2.3. Monovalent Metal Ion Dependency of rN-TcsL UDP-Glucose Hydrolysis Activity

**Figure 4:**
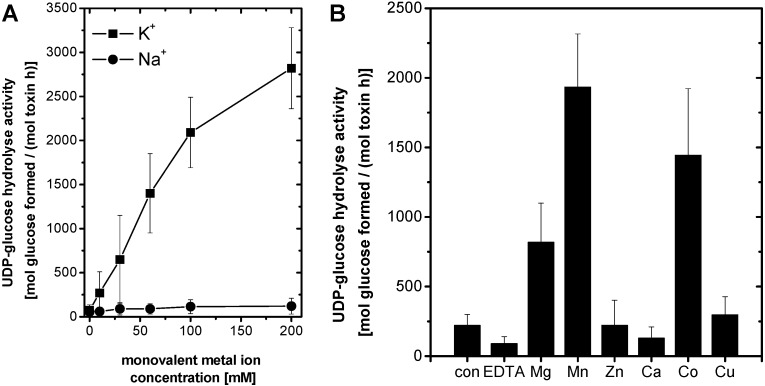
(**A**) K^+^ is an essential co-activator of rN-TcsL. rN-TcsL (100 nM) was incubated with 100 µM·UDP-[^14^C]glucose in the presence of 1 mM·Mn^2+^ and increasing concentrations of K^+^ and Na^+^ as indicated at 37 °C for 30 min. (**B**) Divalent metal ion activation of rN-TcdB. UDP-[^14^C]glucose (100 µM) was incubated in the presence of rN-TcdB (100 nM) with the indicated divalent metal ions (1 mM) at 37 °C for 30 min. UDP-[^14^C]glucose and [^14^C]glucose were separated by thin layer chromatography on PEI cellulose and visualized by autoradiography. Signal intensities of formed [^14^C]glucose were quantified and are given as means ± SD (*n* = 3).

Besides activation by divalent metal ions, several enzymes from eukaryotic and prokaryotic organisms require monovalent metal ions. Monovalent metal ions thereby contribute to anchoring the substrate to the catalytic cleft of the enzyme [23]. To check if rN-TcsL activity depends on monovalent metal ions, UDP-glucose hydrolysis activity was determined in the presence of 1 mM of Mn^2+^ and either KCl or NaCl (Figure 4A). UDP-glucose hydrolysis of rN-TcsL activity was selectively stimulated by increasing concentrations of KCl (not of NaCl) (Figure 4A). Thus, besides activation by divalent metal ions, monovalent K^+^ is an essential co-activator of the rN-TcsL UDP-glucose hydrolysis activity. The requirement for co-activation by K^+^ has also been reported for TcdB and TcdA [18,19]. The intracellular concentrations of K^+^ and Na^+^ range from 100 to 155 mM and from 8 to 30 mM, respectively. LCGTs thus seem to be optimally adapted to the monovalent metal ion conditions in the cytosol of its mammalian target cells. As neither K^+^ or Mg^2+^ alone turn out to be sufficient for rN-TcsL activation, K^+^ and Mg^2+^ may act in tandem to provide optimal docking for the pyrophosphate of the UDP-glucose into the catalytic cleft of the LCGTs, as suggested for several other K^+^-activated enzymes [23].

### 2.4. Metal Ion Dependency of rN-TcdB

TcsL and TcdB share a high identity on the level of amino acids (including the DxD motif) and exhibit comparable 3D structures [16,17]. Re-analysis of the divalent metal ion dependency of rN-TcdB show that the glucosyltransferase (Figure 1) and UDP-glucose hydrolysis activity (Figure 4B) of rN-TcdB were activated by divalent metal ions with a rating: Mn^2+^ > Co^2+^ > Mg^2+^ >> Ca^2+^, Zn^2+^, Cu^2+^. The divalent metal ion dependency of rN-TcdB is highly comparable to that of rN-TcsL. The observations on the rN-TcdB *in vitro* metal ion requirement of UDP-glucose hydrolysis activity is fully consistent with that formerly reported for full-length TcdB [19]. This consistency confirms that rN-TcdB and rN-TcsL are appropriate models for the analysis of the metal ions dependency of the respective full-length toxins.

### 2.5. Prebound Divalent Metal Ions Are Dispensible for Cytopathic Activity of TcsL and TcdB

**Figure 5:**
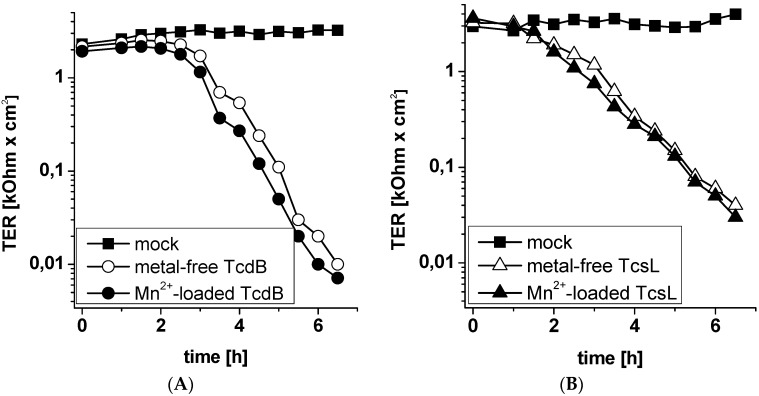
Prebound divalent metal ions are not required for cytopathic activity of TcsL and TcdB. Madin-Darby canine kidney (MDCK-C7) monolayers grown on Transwell filter inserts were treated with TcdB (10 ng/mL, A) and TcsL (30 µg/mL, B). Transepithelial electrical resistance (TER) was monitored for the indicated times as a marker for toxin-uptake and activity. TER values are given as means (*n* = 3).

Cell entry of the LCGTs involves translocation of the N-terminal glucosyltransferase domain from the acid endosome to the cytoplasm by passing through a pore [24,25,26]. This mechanism most likely implies unfolding of the glucosyltransferse domain and the loss of bound factors including metal ions. Refolding of the *N*-terminal glucosyltransferase domain within the cytosol must be hypothesized to recruit intracellular metal ions for correct refolding and intracellular activation of the glucosylation reaction. This hypothesis implies that pre-bound divalent metal ions are not essential for the glucosylation reaction and the cytopathic effects of LCGTs. To corroborate this hypothesis, full-length TcsL and TcdB were depleted from divalent metal ions by incubation with EDTA for 1 h, followed by extensive dialysis against Tyrode’s buffer. The cytopathic effect of TcdB and TcsL was analyzed in terms of the loss of the transepithelial resistance of a Madin-Darby canine kidney (MDCK-C7) monolayer [27]. Divalent metal ion-depleted toxin was either loaded with Mn^2+^ or left untreated and tested on MDCK-C7 monolayers maintained in Tyrode’s buffer. Treatment with divalent metal ion-depleted and Mn^2+^-loaded TcdB and TcsL resulted in a time-dependent loss of the epithelial barrier function of the MDCK monolayer with comparable kinetics, as evidenced in terms of decreasing transepithelial resistance (TER) (Figure 5). This observation suggests that pre-bound metal ions are needed neither for cell entry nor for enzymatic activity. Since divalent metal ion-depleted TcdB and TcsL are inactive (Figure 2B and Figure 4B), the glucosyltransferase domain of either TcdB and TcsL is most likely to recruit intracellular monovalent and divalent metal ions essential for substrate GTPase glucosylation and subsequent cytopathic activity. This view is strongly supported by a recent report showing that the solute carrier family 11 member 1 (SLC11A1), a divalent metal ion transporter, enhances TcdB-catalyzed substrate GTPase glucosylation and TcdB-induced loss of cell viability [28]. Thereby, increased sensitivity of Mn^2+^-pretreated cells to TcdB has been attributed to SLC11A1-dependent Mn^2+ ^transport into the cells [28].

In sum, LCGT-catalyzed GTPase substrate glucosylation requires activation by intracellular monovalent and divalent metals. With regard to the free metal ion concentrations within mammalian target cells, LCGT must be hypothesized to recruit K^+^ and Mg^2+^ for activation of the glucosyltransferase (despite Mn^2+^ being the most efficacious *in vitro* activator).

## 3. Conclusions

The glucosyltransferase and the UDP-glucose hydrolysis activity of TcsL and TcdB require both monovalent and divalent metal ions. The following rating is found for divalent metal ions: Mn^2+^ > Co^2+^ > Mg^2+^ >> Ca^2+^, Cu^2+^, Zn^2+^.

TcsL and TcdB share comparable monovalent and divalent metal ions requirement, consistent with comparable 3D structures of their glucosyltransferase domains.

TcsL and TcdB most likely recruit intracellular K^+^ and Mg^2+^ for activation of the glucosyltransferase.

## 4. Materials and Methods

Materials—All reagents were of analytical grade and purchased from commercial sources. The glucosyltransferase domain (covering amino acids 1 to 546) of TcsL (rN-TcsL), TcdB (rN-TcdB), and Rac1 were recombinantly expressed in *E. coli* and purified as GST fusion proteins as described [20]. TcsL and TcdB were prepared from *C. sordellii* strain 6018 and *C. difficile* strain VPI10463, respectively, as previously described [15,29].

Transepithelial resistance of MDCK-C7 monolayers—Madin-Darby canine kidney (MDCK-C7) cells were cultured under standard conditions (37 °C, 5% CO_2_) as described [27]. Briefly, MDCK-C7 cells were cultured in minimum essential medium (MEM) enriched with Earle’s salts, non-essential amino acids, glutamic acid, and 10% fetal calf serum (Biochrom, Berlin, Germany) and split twice weekly using standard culture techniques. MDCK-C7 cells were seeded onto 12-well filter transwell inserts (pore size 0.4 µM, Becton Dickinson, Heidelberg, Germany). The transepithelial electrical resistance (TER) was determined by a Voltohmmeter equipped with Endom 24 chamber (EVOM, World Precision Instruments, Berlin, Germany). MDCK-C7 monolayers were cultivated up to an initial resistance of > 2 kΩ·cm^2^. The medium was exchanged for HEPES-buffered Tyrode’s solution (25 mM of HEPES, pH 7.4, 120 mM·NaCl, 5 mM·KCl, 2 mM·CaCl_2_, and 6 g/L of glucose). The toxins were applied on the basolateral site of the monolayer and toxin-induced loss of TER was analyzed in a time-dependent manner.

Glucosyltransferase activity—Recombinant Rac1 (50 µg/mL) was incubated with either rN-TcsL or rN-TcdB, 20 µM of UDP-[^14^C]glucose (Biotrend, Cologne, Germany), and the indicated divalent metal ions (1 mM) in glucosylation buffer (50 mM of HEPES pH 7.4, 0.1 mM·MgCl_2_, 150 mM·KCl, 100 µg/mL of BSA) in a total volume of 20 µL at 37 °C for 10 min. Proteins were analyzed by 12.5% SDS-PAGE, and [^14^C]glucosylated Rac1 was visualized by PhosphorImaging (Cyclone, PerkinElmer Life and Analytical Sciences, Shelton, CT, USA).

Glycohydrolase activity—The toxins were incubated with 100 µM of UDP-[^14^C]glucose in glycohydrolase buffer (50 mM of HEPES, pH 7.4, supplemented with either 150 mM·KCl or NaCl, and 100 µg/mL of BSA) in the presence of the indicated concentrations of divalent metal ions. Samples of a total volume of 10 µL were incubated at 37 °C for 30 min. 2 µL samples were taken as indicated and run on PEI-cellulose plates (Merck, Darmstadt, Germany) using 0.2 mM·LiCl as mobile phase. The plates were dried and analyzed using PhosphorImager. Quantitative data are given as mol formed [^14^C]glucose per mol toxin and per time indicated as means ± SD (*n* = 3).

Data analysis—Initial rate data for the glycohydrolase reaction were determined with regard to metal ion binding by varying the metal ion concentration. The EC_50_ values were obtained by a non-linear curve fitting using the sigmoid E_max_ model (the Hill equation).

## Abbreviations

The following abbreviations are used in this manuscript:

LCGT	Large clostridial glycosylating toxin
PEI	Polyethylenimine-modified
TcdA	*C. difficile* toxin A
TcdB	*C. difficile* toxin B
Tcnα	*C. novyi* alpha-toxin from, and the glycosylating toxin
TcpE	Glycosylating toxin from *C. perfringens* type E strain
TcsL	*C. sordellii* lethal toxin
TcsH	*C. sordellii* hemorrhagic toxin
